# Investigating bisulfite short-read mapping failure with hairpin bisulfite sequencing data

**DOI:** 10.1186/1471-2164-16-S11-S2

**Published:** 2015-11-10

**Authors:** Jacob Porter, Ming-an Sun, Hehuang Xie, Liqing Zhang

**Affiliations:** 1 Department of Computer Science, Virginia Tech, Blacksburg, 24061, VA, USA; 2 Virginia Bioinformatics Institute, Virginia Tech, 24061 Blacksburg, VA, USA; 3 Department of Biological Sciences, Virginia Tech, 24061 Blacksburg, VA, USA

**Keywords:** Methylation, Bisulfite short read mapping, Next-generation Sequencing, hairpin bisulfite data, Sequence Complexity, Entropy

## Abstract

**Background:**

DNA methylation is an important epigenetic mark relevant to normal development and disease genesis. A common approach to characterizing genome-wide DNA methylation is using Next Generation Sequencing technology to sequence bisulfite treated DNA. The short sequence reads are mapped to the reference genome to determine the methylation statuses of Cs. However, despite intense effort, a much smaller proportion of the reads derived from bisulfite treated DNA (usually about 40-80%) can be mapped than regular short reads mapping (> 90%), and it is unclear what factors lead to this low mapping efficiency.

**Results:**

To address this issue, we used the hairpin bisulfite sequencing technology to determine sequences of both DNA double strands simultaneously. This enabled the recovery of the original non-bisulfite-converted sequences. We used Bismark for bisulfite read mapping and Bowtie2 for recovered read mapping. We found that recovering the reads improved unique mapping efficiency by 9-10% compared to the bisulfite reads. Such improvement in mapping efficiency is related to sequence entropy.

**Conclusions:**

The hairpin recovery technique improves mapping efficiency, and sequence entropy relates to mapping efficiency.

## Background

DNA methylation is an epigenetic phenomenon that adds a methyl group to the nucleic acid cytosine in DNA. Methylation impacts evolution and inheritance [1], embryonic development [2,3], and cancer [4,5]. Discovering where DNA methylation occurs can be accomplished with the sequencing of bisulfite treated DNA. Bisulfite transforms unmethylated cytosine into uracil. The methylated cytosine sterically hinders the sulfite ion preventing transformation of methylated cytosine into uracil. The DNA is amplified with polymerase chain reaction (PCR) where the uracil is converted to thymine. Differences between a reference genome and the bisulfite treated DNA can be used to search for methylated cytosines.

To identify such differences, the most important step is to map the bisulfite treated short reads to the reference genome. Mapping software takes a DNA sequence and finds a matching position for it in a reference genome. Many mapping programs suitable for bisulfite treated short reads exist. These include Bismark [6], BSMap [7], BiSS from NGM [8], BatMeth [9], and BS-Seeker2 [10]. These programs work by first finding a potential location for the read using hashing or a string index. The next step is to compute a score for the read.

This can be done by counting mismatches and indels. Some programs use the Smith-Waterman algorithm [11] or the Needleman-Wunsch algorithm [12]. The best scoring read location is reported as the location for that read. Despite continued and intense effort in creating new or improving existing bisulfite read mappers, the proportion of mapped reads remains low, commonly around 40% [6,13], and sometimes as high as 80% especially with read trimming [9,13], which is much lower than regular short reads mapping (e.g., data for the 1000 Genomes project has mapping efficiency > 90% [14]). It is unclear what leads to the reduced efficiency in bisulfite reads mapping.

**Figure 1:**
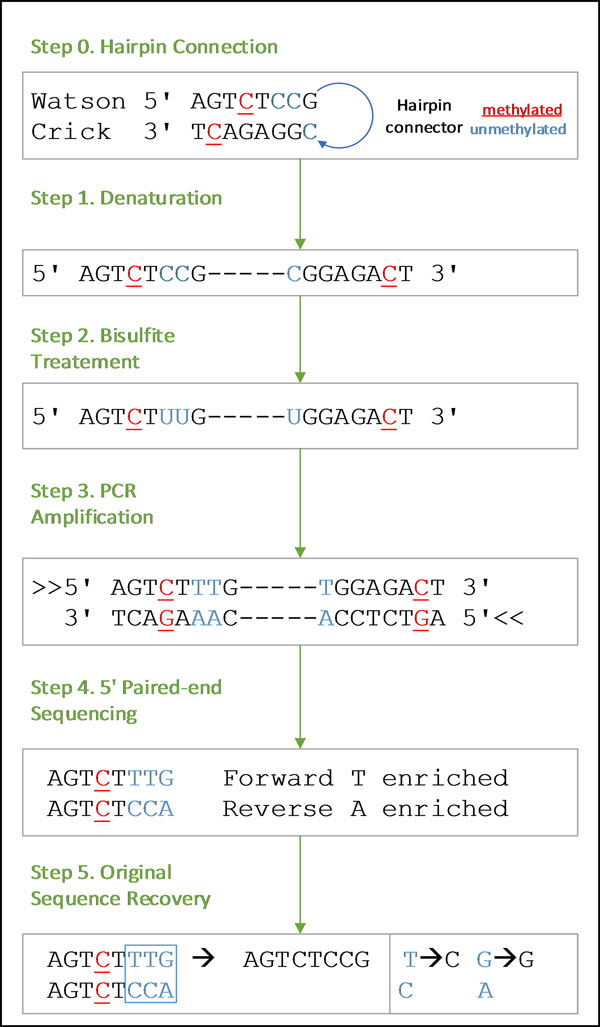
**Example of bisulfite-treated hairpin PCR sequencing with recovery of the original sequence**.

In this work, we use a sequencing strategy called genome-wide hairpin sequencing of bisulfite-treated short reads to investigate factors that may adversely influence mapping efficiency of bisulfite reads [15]. Unlike previous bisulfite sequencing methods that destroy knowledge of the original (non-bisulfite treated) sequence, the hairpin technology (see Figure 1) allows for the recovery of the original sequences by putting a connector between the Watson and Crick strands and then using PCR and paired end technology to sequence short reads [16]. The resulting sequences give paired strands that can be mapped to recover the original untreated read and to detect sequencing error.

This study focuses on the read mapper Bismark, which is popular, easy to use, and reasonably accurate [13]. It converts all Cs to Ts in the read and the reference genome, and then calls Bowtie2 as a sub-process to do the mapping. Bowtie2 uses an FM-Index, a compressed string index, of the reference genome to find read positions on the reference genome. Bowtie2's scoring function weights mismatches, indel starts, and indel extensions. It can report the score for the best mapping as well as the next best mapping [6,17]. Because the hairpin method enables recovery of the original sequences from bisulfite converted reads, the effects of bisulfite treatment on a mapping program can be studied by mapping bisulfite converted reads with Bismark and recovered (original) reads with Bowtie2. This isolates bisulfite treatment as the only variable affecting mapping quality. We also used the bisulfite mapper BSMap, one of the first bisulfite mapper programs, for comparison. It creates a hash table of the reference genome of all possible C to T conversions in a sliding window of 16 base pairs in the reference genome [7]. The following have been published by the same authors in an extended abstract: some results on original sequence recovery and entropy, most of the methods section, and Figure 1[18].

## Methods

### Genome-wide mouse hairpin bisulfite-sequencing data creation

We first generated genome-wide hairpin bisulfite-sequencing data for mouse ES cells (E14TG2a) using the technology developed by our lab (GEO accession number GSE48229) [15]. To induce differentiation, the mouse ES cells were cultured in ES cell culturing media for six days without LIF (Leukemia inhibitory factor). Genomic DNA isolated from the undifferentiated (E14-d0) and differentiating (E14-d6) states of mES cells were used for library construction. Briefly, after DNA sonication, end repair, and dA tailing, the DNA fragments were further ligated to Biotin-modified hairpin adapter and Illumina TruSeq adapters. Adapter-ligated DNA was digested with MseI and MluCI (NEB) to enrich regions with high CpG density, and then pulled down using Dynabeads ^® ^MyOneTM Streptavidin C1 beads (Invitrogen). After bisulfite conversion and PCR, size selection of 400-600 bp fragments was conducted to yield longer sequences that are more amenable for unambiguous mapping to the reference sequence.

### Unconverted sequence recovery

Figure 1 shows how the bisulfite treated hairpin PCR sequencing technology works and how the original non-bisulfite sequence can be recovered. In step 0, the hairpin connector is attached to opposing Watson and Crick strands. The opposing strands are denatured in step 1 and then treated with bisulfite in step 2. Bisulfite treatment converts un-methylated cytosine to uracil. Step 3 involves PCR amplification, which copies the forward and reverse sequences and converts uracil into thymine. In step 4, paired-end sequencing technology is used to sequence from the 5' ends. This gives a sequence pair. Notice that one strand is enriched for Ts since the PCR and bisulfite treatment converts unmethylated Cs to Ts. The other strand is enriched for As since the Crick strand's un-methylated Cs become Ts, which are complemented with As. These As are then paired with Gs in the opposing strand. Finally, step 5 recovers the original sequence. When the T-enriched strand has a T and the A-enriched strand has a C, this maps to a C, and when the T-enriched strand has a G and the A-enriched strand has an A, this maps to a G. All other mismatches are due to errors in PCR or sequencing since the two strands should match up perfectly as they come from opposing sequences in the DNA.

### Software installation and use

All software development and analysis was performed on the bioinformatics clusters hosted by the Virginia Bioinformatics Institute and the Virginia Tech CS department. Two of the machines consist of two quad core processors (Intel(R) Xeon(R) CPU E5-2407 0 @ 2.20 GHz) each with 128 gigabytes of memory. Two other machines consisted of three eight core processors (Intel(R) Xeon(R) CPU E5-2660 0 @ 2.20 GHz) each, where one machine has 128 GB of memory and the other has 192 GB of memory. Bowtie2 (version 2.1.0) was compiled with the Gnu Compiler Collection. Bismark (version 0.7.12) was run with Perl (version 5.10.1) with the non-directional flag and default settings. Blastn (version 2.2.28+) was run with default settings on a C-to-T converted reference genome to map unmapped Bismark reads, where all of the reads had Cs converted to Ts. Bismark reports unique mappings, which are mappings that have a unique best score, ambiguous mappings, which are reads with locations that have multiple best scores, and unmapped reads, which had scores that were too low or reads where a location couldn't be assigned.

### File processing and statistical calculation

Custom Python 2.7 [19] scripts using BioPython [20] were created to process files and calculate statistics such as sequence entropy and sequence length. File manipulation included randomly sampling from FASTA files.

Sequence entropy was calculated for each sequence in each of the mapping categories (unique, ambiguous, and unmapped). Entropy for a sequence, s, is calculated by taking the negative sum of the frequency of each base, *f_b_*, times the logarithm of the frequency of each base. More formally,

$$Ent\left(s\right)=-\underset{b\in \left\{A,C,T,G\right\}}{\sum }f_{b}\text{log}\left(f_{b}\right)$$

Entropy gives a measure of sequence randomness [21]. This measure of sequence complexity was chosen since it is simple and fast to compute. A sequence of all Ts will have an entropy of 0, but a sequence with 25 percent of each base will have an entropy of 2. We hypothesized that entropy would affect mapping quality. Highly random-seeming sequences should uniquely map while non-random sequences, for example, a sequence with mostly Ts, will map ambiguously or be discarded since the string of Ts could map to many portions of the genome that have Cs at indeterminate locations in the string of Ts. Bisulfite treatment tends to reduces entropy since most C's will be converted to T's since cytosine methylation is usually rare.

For comparing the unique mapping efficiency for converted and unconverted reads, 50 bootstrap replicates with replacement were created from all of the mouse data (both differentiating and undifferentiated). Each replicate had 6.46 million reads. Each replicate had the T-enriched pair and the A-enriched pair so that the untreated original sequence could be recovered. Reads that aligned exactly (that is, there was no sequencing error) were extracted and mapped with Bismark on default settings for both the T-enriched and the A-enriched reads. About 68% (4.4 million) of the reads in each replicate aligned exactly. The original read was recovered and mapped with Bowtie2 using the same settings as Bismark. Bismark reports a uniquely mapped percent, and the recovered sequence mapping categories from Bowtie2 was used to calculate a uniquely mapped percent in the same way as Bismark. Read mappings were done for all 50 bootstrap replicates so that a p-value could be computed.

For a single bootstrap replicate for the day 0 data, reads were extracted that had 0, 1, 2, and 3 mismatches somewhere in the read. Mapping using Bismark was done for all of them. Bismark mappings for reads with mismatches only in the first 25 bases were compared with reads with mismatches only in the latter right-most part. This comparison was done for 1, 2, and 3 mismatches.

With Illumina pair-end sequencing technology, a total of 8 "lanes" of data was produced. Lane 8 data was used throughout except in the two cases mentioned above that used bootstrap replicates drawn from all of the data. Both T-enriched and A-enriched strands were used for analysis, and the day 0 and day 6 data was used. The day 0 lane 8 data has 32,434,798 reads and the day 6 lane 8 data has 58,034,817 reads.

To understand what the best possible bisulfite mapping efficiency was, a simulation was performed where simulated bisulfite reads were sampled from the mouse reference genome at random and read sequencing error was introduced. This simulation represented the best possible mapping scenario since the reads did not include natural variation. The simulation sampled sequences of length 100, 75, and 50 bp in the same proportions that were in the lane 8 data and compared mapping efficiency to the real hairpin data with sequences of only 100, 75, and 50 bases. Simulations with sequencing error of 1% and 10% were created. (Sequencing error set to less than 1% had very little difference in the mapping efficiencies of 1% error.) The program Sherman from Babraham Bioinformatics was used to do the simulation with methylation rates set to CpG = 80% and CPH = 2%. These rates were chosen since they were consistent with the literature [15]. Both BSMap and Bismark were used to do the mapping.

To investigate the benefit of using different algorithms, Blast was run on default settings on 64 percent of the bisulfite-treated reads unmapped by Bismark for the T-enriched day 0 lane 8 data. The reads and the reference genome had all C's converted to T's. Only 64 percent of the reads were used since Blast took a long time to complete and produced a lot of data.

## Results and discussion

### Bisulfite treated reads have low mapping efficiency

Bismark uniquely mapped 47.5-52 percent of the bisulfite converted reads in the mouse data, ambiguously mapped 27-34 percent, leaving 16-20 percent unmapped. Therefore, around half of the reads don't give a strong signal for their position, rendering them less biologically useful.

### Sequence entropy relates to mapping efficiency

**Figure 2:**
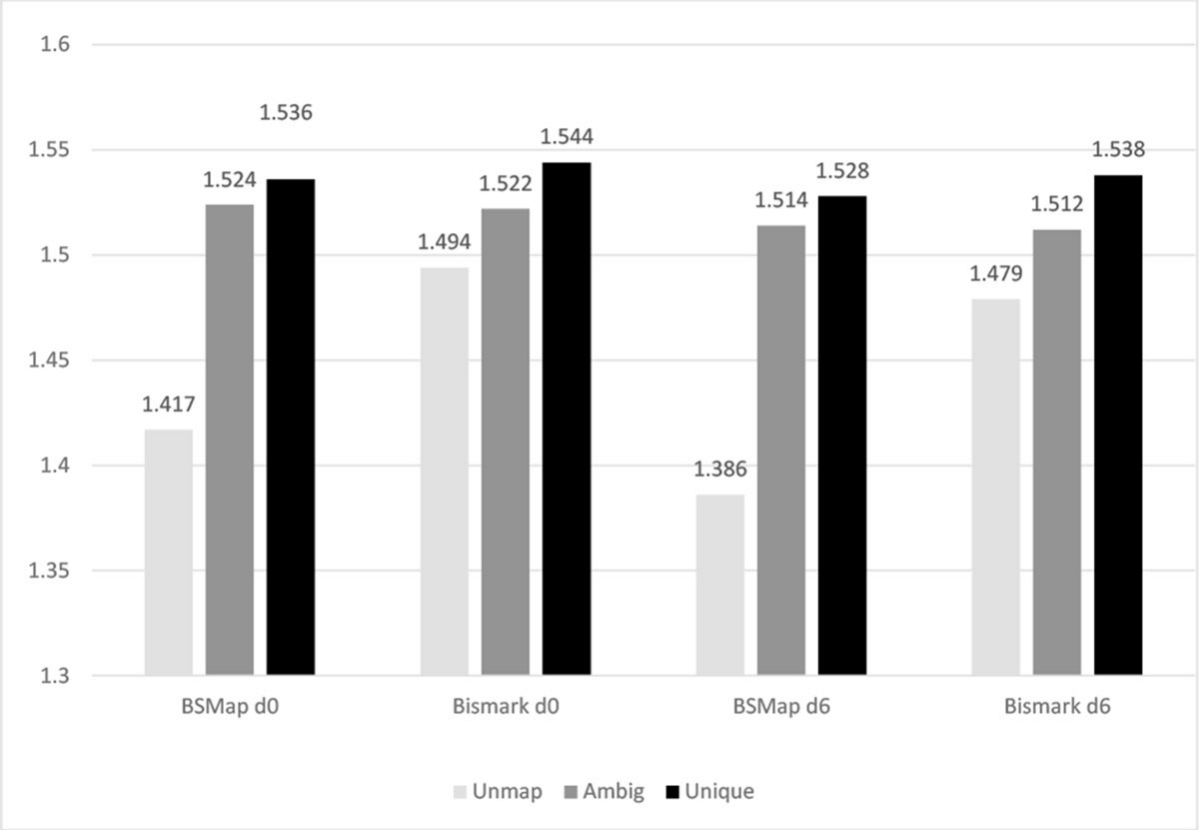
**Bismark and BSMap average sequence entropy by mapping category for both day 0 and day 6 data**.

We examined the average sequence entropy by mapping category for both day 0 and day 6 data when mapped with Bismark or BSMap (Figure 2). Both mappers show a trend of increasing average entropy when going from unmapped to ambiguous to unique mappings.

**Figure 3:**
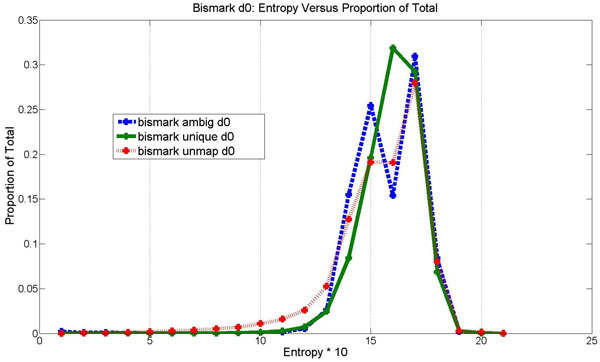
**Bismark entropy distribution on day 0 data by mapping category: ambiguous, unique, unmapped**. The x-axis is entropy times 10, and each integer point × is a bucket of sequences where × represents sequences with entropy values between x/10 and (x-1)/10. The y-value is the proportion of the total number of sequences in the mapping category.

Figure 3 shows a histogram of sequence entropy by mapping category for the hairpin data on day 0 mapped with Bismark. There is a spiking phenomenon around 1.5 that may correspond with reduced entropy due to bisulfite treatment. During bisulfite conversion, most of the Cs were converted to Ts and thus the frequency of the Cs is greatly reduced. In this distribution, the maximum differences between the mapping categories were determined as the following: unmap-ambig: 0.05, unmap- unique: 0.12, unique-ambig: 0.17. The Kolmogorov-Smirnoff two-sample tests were performed and the differences between these distributions are with p-values less than 0.01. Because uniquely mapped reads tend to have higher entropy, entropy positively correlates with improved mapping efficiency. This suggests that reduced entropy from bisulfite treatment contributes to worsened mapping effectiveness.

### Recovering the original sequence improves mapping efficiency

We hypothesized that the hairpin sequencing technology can improve the mapping efficiency by recovering the original sequences, which have higher entropy. For the undifferentiated data, recovering the untreated sequence improved unique mapping efficiency by an average of 9.15% (p-value = 0, variance≈ 10^−8^) compared to the average unique mapping efficiency of the T-enriched and the A-enriched reads. For the differentiating data, the unique mapping efficiency was improved by 10.59% (p-value = 0, variance ≈ 10−8). This shows the benefit of recovering the information lost from bisulfite treatment and is consistent with the notion that more entropic reads map better.

### Different hairpin sequences may map differently

We next checked whether mapping efficiency can be improved without sequence recovery but with mapping information from additional sequence read, since one sequence from the hairpin sequences may uniquely map while the other doesn't. Bismark was run on the lane 8 data for both day 0 and day 6. Both the T-enriched and the A-enriched sequences were compared to see if one sequence uniquely mapped while the other didn't. If one sequence uniquely maps, then the other sequence can be considered to uniquely map as well. This improves the overall mapping efficiency. For the day 0 data, about 19 percent of the T-enriched sequences that uniquely mapped did not uniquely map in the A-enriched sequences, and vice-versa. For the day 6 data, about 22 percent of the T-enriched sequences that uniquely mapped did not uniquely map in the A-enriched sequences, and vice-versa. This suggests that the hairpin sequencing strategy can improve mapping efficiencies by uniquely mapping one sequence to find the position of the other sequence.

### Sequencing error adversely affects mapping efficiency

**Figure 4:**
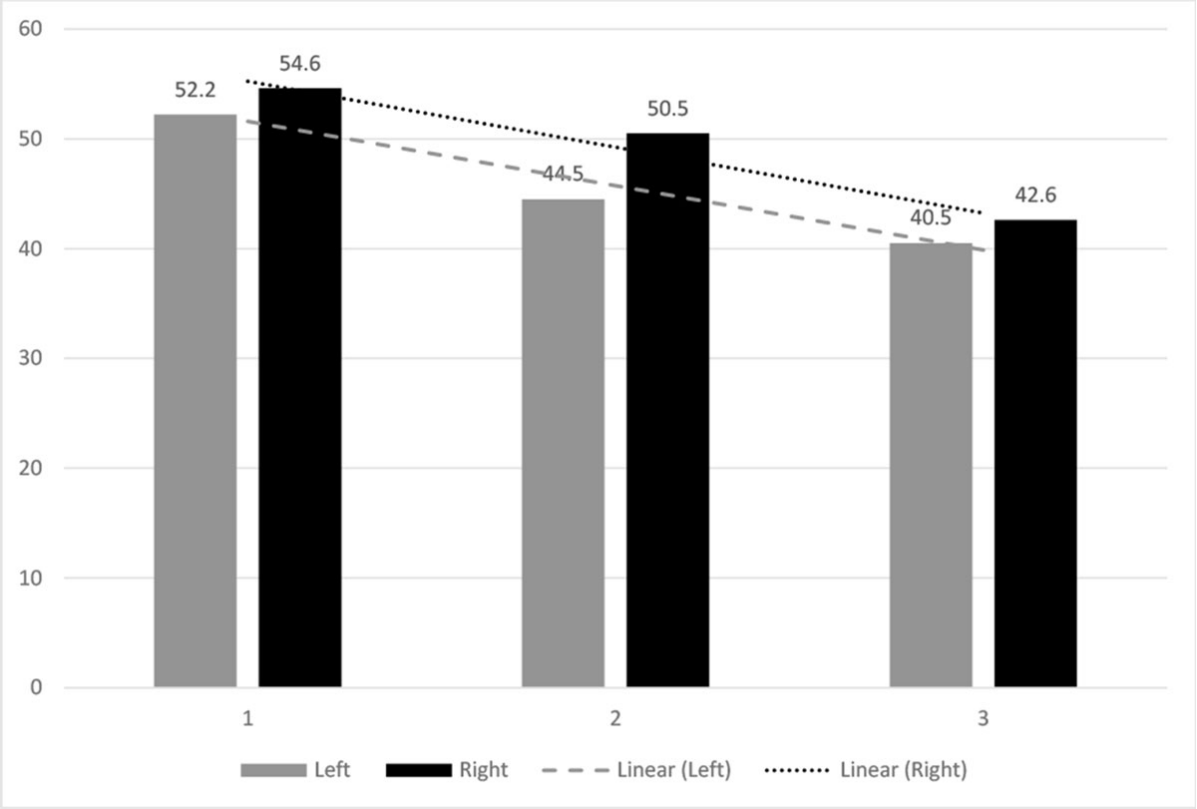
**Number of mismatches on the left or the right side of the read versus alignment efficiency with day 0 data with Bismark unique alignment efficiency**. The left side is the first 25 (5' end) bases of the read. The right side is the other bases.

The number of mismatches in reads could be determined by mapping two pair-end sequences together. We observed a downward trend in mapping efficiency with increasing mismatch count (Figure 4). Figure 4 shows the results of mapping reads with mismatches in the first 25 bases compared with mapping reads with mismatches only in the other bases. The unique mapping efficiency is consistently lower when mismatches are in the first 25 bases, and it is worsened with increased mismatch count. Thus, mismatches correlate with low mapping efficiency, especially in the 5' end. The hairpin sequencing technology can be used to identify mismatch areas of a read for removal so that unique mapping efficiency can be increased.

### Bismark and BSMap mappings are highly overlapping

The T-enriched sequences for the day 0 data were mapped with Bismark and BSMap. Combining the uniquely mapped sequences of both mappers (an OR operation) resulted in only about a 2 percent increase over BSMap alone. Other work has shown greater differences between these programs for other data [13]. Therefore using multiple mappers may improve mapping efficiencies. Using multiple mappers increases the confidence that a read is mapped correctly when the mappers map to the same location.

### Longer sequences tend to map uniquely

Reads in the mouse hairpin data ranged from 40 to 101 bases. Day 0 lane 8 data had 63.4 percent reads with lengths either 100 or 101, and day 6 lane 8 data had 69.34 percent reads with 100-101 bases. We suspected that longer nucleotide sequences would have higher unique mapping efficiency since the probability that a longer sequence is repeated in the genome is low. For example, the sequence consisting of only "A" is often found, but the sequence "ATCGGTGCCAT" will be found less often.

**Figure 5:**
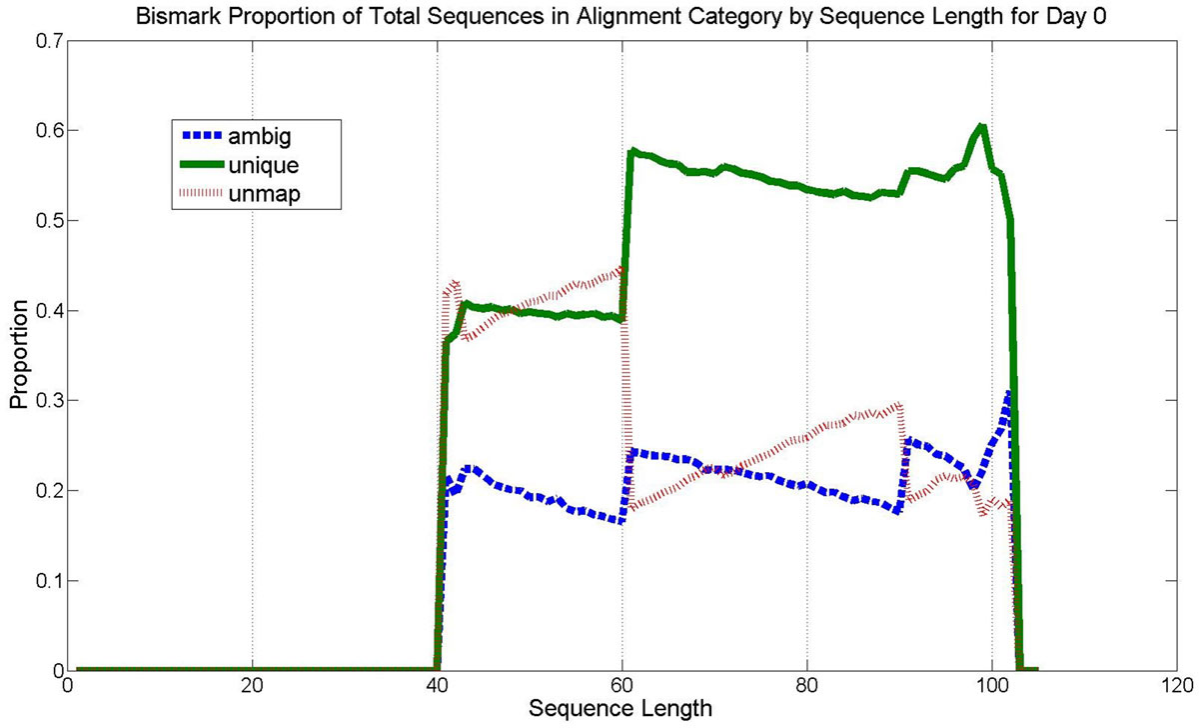
**Bismark mapping categories by sequence length on day 0 data**. This shows mapping efficiency by length for the day 0 lane 8 data for each mapping category: unique, ambiguous, and unmapped.

The programs BSMap and Bismark were used to do mapping, and then the percentage of reads of a given length that belong to each mapping category was calculated as can be seen in Figure 5 for Bismark. For Bismark, there is a generally upward trend with longer sequences more often mapping uniquely and longer sequences less often unmapped. There are noticeable spikes at lengths 60 and 90. However, BSMap's mapping efficiency remains about flat (no spiking) until longer reads are mapped where there is a slight increase in mapping efficiency. These results confirm the hypothesis that longer reads tend to map uniquely.

### Sequencing error simulation suggests an upper bound on mapping performance

**Figure 6:**
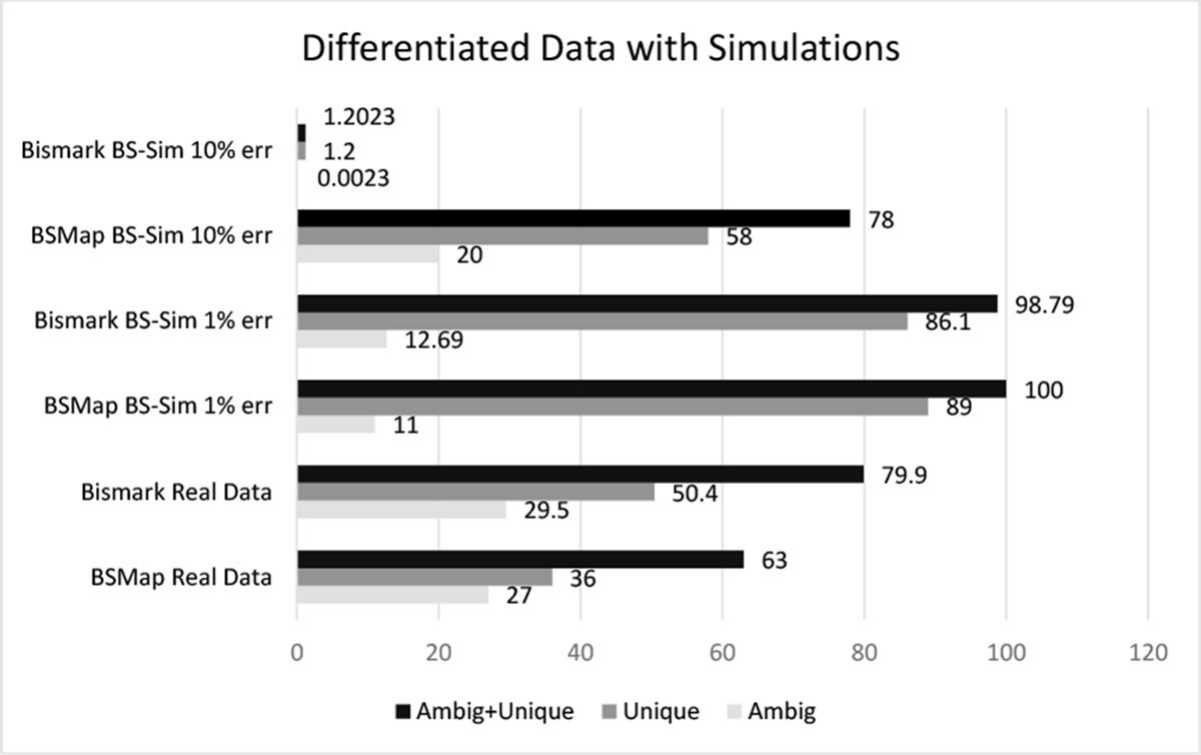
**Mapping results for both simulated and real data on sequences of length 100, 75, and 50 distributed in the same way as the hairpin data set**. Simulation on bisulfite treated at 1% and 10% error rate. The mapping was done with BSMap and Bismark.

Figure 6 shows that with one percent sequencing error and with reads drawn randomly from the mouse reference genome, Bismark uniquely maps 86.1 percent of the reads and ambiguously maps 12.7 percent of the reads. The best possible mapper probably won't be able to do much better than this. This compares to 50.4 percent unique mapping efficiency by Bismark on bisulfite treated data according to Figure 6. This means that read mappers could improve by over 30 percent. A high sequencing error of 10% causes an almost complete failure of Bismark, and BSMap worsens by 22%.

### Blasting unmapped reads produces hits

Last, we investigated the benefit of using different algorithms for mapping by using Blast to map reads unmapped by Bismark. Blast significantly mapped around 30 percent of these unmapped reads. Blast is noticeably slower and its output is not in a convenient format for variant calling; nonetheless, this result shows that more computationally intense methods improve mapping efficiency.

## Conclusions

In this study, we observed low sequence entropy co-occurs with low unique mapping efficiency, and high entropy co-occurs with high unique mapping efficiency. This suggests that lost entropy from bisulfite treatment causes worse mapping efficiency. In addition, mismatches in the seed region for a mapper correlate with worsened performance. The hairpin sequencing strategy is useful in improving mapping performance of bisulfite treated reads. With sequence information from two DNA strands, the original sequence can be recovered and PCR or sequencing error mismatches can be identified. Hairpin data can be used to ameliorate lost entropy by recovering the original sequence and improving unique mapping efficiencies.

Additional coping strategies involve using multiple read mappers or more computationally intense software such as Blast to improve mapping results since they can be used to map reads that are unmapped by less computationally intense software such as Bismark and Bowtie2.

## Competing interests

The authors declare that they have no competing interests.

## Authors' contributions

JP conceived and carried out most of the data analysis and wrote the scripts, and he wrote much of the manuscript. MS prepared the DNA bisulfite hairpin data and contributed to the writing pertaining to data preparation. HX supervised the data preparation and contributed to the manuscript. LZ contributed to the manuscript and the conception of the data analysis.

## Declarations

Publication of this article was funded by the VBI new faculty startup fund for HX and Virginia Tech's Open Access Subvention Fund.

This article has been published as part of *BMC Genomics *Volume 16 Supplement 11, 2015: Selected articles from the Fourth IEEE International Conference on Computational Advances in Bio and medical Sciences (ICCABS 2014): Genomics. The full contents of the supplement are available online at http://www.biomedcentral.com/bmcgenomics/supplements/16/S11.
